# Novel technologies in doubled haploid line development

**DOI:** 10.1111/pbi.12805

**Published:** 2017-09-11

**Authors:** Jiaojiao Ren, Penghao Wu, Benjamin Trampe, Xiaolong Tian, Thomas Lübberstedt, Shaojiang Chen

**Affiliations:** ^1^ National Maize Improvement Center of China China Agricultural University Beijing China; ^2^ Department of Agronomy Iowa State University Ames IA USA; ^3^ College of Agronomy Xinjiang Agriculture University Urumqi China

**Keywords:** doubled haploid, haploidization, chromosome elimination, genome doubling, minichromosome

## Abstract

haploid inducer line can be transferred (DH) technology can not only shorten the breeding process but also increase genetic gain. Haploid induction and subsequent genome doubling are the two main steps required for DH technology. Haploids have been generated through the culture of immature male and female gametophytes, and through inter‐ and intraspecific via chromosome elimination. Here, we focus on haploidization via chromosome elimination, especially the recent advances in centromere‐mediated haploidization. Once haploids have been induced, genome doubling is needed to produce DH lines. This study has proposed a new strategy to improve haploid genome doubling by combing haploids and minichromosome technology. With the progress in haploid induction and genome doubling methods, DH technology can facilitate reverse breeding, cytoplasmic male sterile (CMS) line production, gene stacking and a variety of other genetic analysis.

## Introduction

Based on the 2015 *Revision of World Population Prospects*, the world population will reach 9.7 billion in 2050 (https://esa.un.org/unpd/wpp/). Feeding the growing population in 2050 is estimated to require increasing overall food production by 70% (http://www.fao.org/wsfs/forum2050/wsfs-forum/en/). With limited natural resources, land and water, and the challenges of a changing climate, the yield of major food crops, maize, rice and wheat, needs to be increased over time. Continued increases in crop performance can be obtained by steeper genetic gains mediated by improved marker technologies, predictive statistics and breeding methodologies (De La Fuente *et al*., 2013). Genetic gain (*Δ*_*G*_) depends on additive genetic (*σ* ^2^_*A*_) and phenotypic variance (σ^2^_*p*_), selection intensity (*k*), parental control (*c*) and the number of years required per generation (*Y*). The equation for genetic gain per year is ▵_*G*/*Y*_=*kc*σ^2^_*A*_/*Y*σ_*p*_ (Dwivedi *et al*., 2015). Doubled haploid (DH) technology has provided a strategy to significantly shorten breeding cycles and increase genetic gain.

The major advantage of DH technology lies in instantaneous development of homozygous lines instead of 6–10 generations of inbreeding by selfing or sib‐crossing (Prigge *et al*., 2012), which is a major breakthrough to speed up cultivar development (Dunwell, 2010). DH has been discovered in at least 200 plant species and is widely used in Brassicas and cereals, including wheat, barley, rice and maize (Dunwell, 2010; Dwivedi *et al*., 2015; Forster *et al*., 2007; Germanà, 2011). Haploids are generated by *in vitro* procedures based on the culture of immature male and female gametophytes and by *in vivo* procedures based on inter‐ and intraspecific hybridization causing uniparental chromosome elimination. Once haploid plants become available, their genome must be doubled to produce a fertile DH line (n→2n). In some species, inefficient haploid genome doubling is considered to be the key obstacle for implementation of DH technology in commercial breeding programmes. Genes and quantitative trait loci (QTL) involved in haploid induction and genome doubling have been reported (Table 1). This opinion paper focuses on haploidization via chromosome elimination, especially recent advances in centromere‐mediated haploidization, haploid genome doubling, particularly the improvement of genome doubling with minichromosome technology, and application of DHs to accelerate plant breeding and genetic analyses.

**Table 1:** Genes/QTL and their function with regard to haploid induction and doubling

Category	Name	Function	Species	Reference(s)
Gene	*MATRILINEAL (MTL)*/ *ZmPLA1*/ *NOT LIKE DAD (NLD)*	Sperm‐specific phospholipase triggers maize haploid induction	Maize	Kelliher *et al*. (2017), Liu *et al*. (2017), Gilles *et al*. (2017)
Gene	*indeterminate gametophyte* (*ig*)	An LOB domain protein affects haploid induction	Maize	Evans (2007)
Gene	haploid initiator gene (*hap*)	Prevents fertilization of the egg cell and not affecting fertilization of the polar nuclei and development of the endosperm	Barley	Hagberg and Hagberg (1980), Hagberg and Hagberg, (1981), Mogensen (1982)
Gene	*CENH3*	Haploid induction through centromere‐mediated chromosome elimination	Arabidopsis	Ravi and Chan (2010)
Gene	*first division restitution 1 (fdr1)*	Restores haploid male fertility attributable to first division restitution and produce diploid kernels	Maize	Sugihara *et al*. (2013)
Gene	*MiMe* genotype (*osd1*/*spo11‐1*/*rec8*)	Transfers meiosis into mitosis	Arabidopsis	Cifuentes *et al*. (2013)
QTL	*qhir2‐qhir8*	Haploid induction	Maize	Liu *et al*. (2015), Prigge *et al*. (2012)
QTL	*qmhir1* and qmhir2	Maternal genetic effect of haploid induction	Maize	Wu *et al*. (2014)
QTL	*qhmf1‐ qhmf4*	Haploid male fertility	Maize	Ren *et al*. (2017)

## Haploidization via chromosome elimination

### Haploidization via interspecific hybridization

Haploids can be obtained from the progeny of crosses between parents from different species by a process of selected chromosome elimination (Forster *et al*., 2007; Kasha and Kao, 1970; Laurie *et al*., 1990; Wędzony *et al*., 2009). This process was first discovered in barley (*H. vulgare* × *H. bulbosum)* (Kasha and Kao, 1970). Hybrids with both sets of parental genomes can be obtained after pollination (Humphreys, 1978), followed by selective loss of *Hordeum bulbosum* chromosomes soon after (Bennett *et al*., 1976; Gernand *et al*., 2006; Kasha and Kao, 1970; Symko, 1969). This method has been quickly improved and standardized by researchers and termed ‘The *Hordeum bulbosum* (L.) method' (Devaux, 2003). Later, wheat haploids produced by wheat × maize hybridization were reported (Laurie and Bennett, 1986). In the wheat × maize system, hybrid embryos are generated, but maize chromosomes are soon after effectively eliminated to form haploid wheat embryos. Maize is also the most popular pollen donor for haploid induction in other cereals, like triticale, rye and oats (Immonen and Tenhola‐Roininen, 2003; Rines, 2003; Wędzony, 2003). The interspecific hybridization method was also reported to induce haploids in additional species crosses, such as wheat × pearl millet (Laurie, 1989), pear × apple (Inoue *et al*., 2004) and *Triticum aestivum* × *Triticeae* species (Liu *et al*., 2014). Genetic and environment conditions, such as temperature and light intensity, may affect haploid frequency (Bitsch *et al*., 2000; Campbell *et al*., 2001; Garcia‐Llamas *et al*., 2004; Sanei *et al*., 2011).

The mechanisms underlying selective chromosome elimination following interspecific pollinations are in most cases unknown. Generally, double fertilization leads to hybrid zygotes. However, during early embryogenesis, uniparental chromosome elimination results in haploid embryos (Forster *et al*., 2007). Chromosome elimination also happens in rapidly dividing endosperm leading to abortion in seed development. Differences in timing of mitotic processes due to asynchronous cell cycles, parent‐specific inactivation of centromeres, asynchrony in nucleoprotein synthesis and many other hypotheses have been put forward to explain selective chromosome elimination (Gernand *et al*., 2005; Sanei *et al*., 2011).

**Figure 1:**
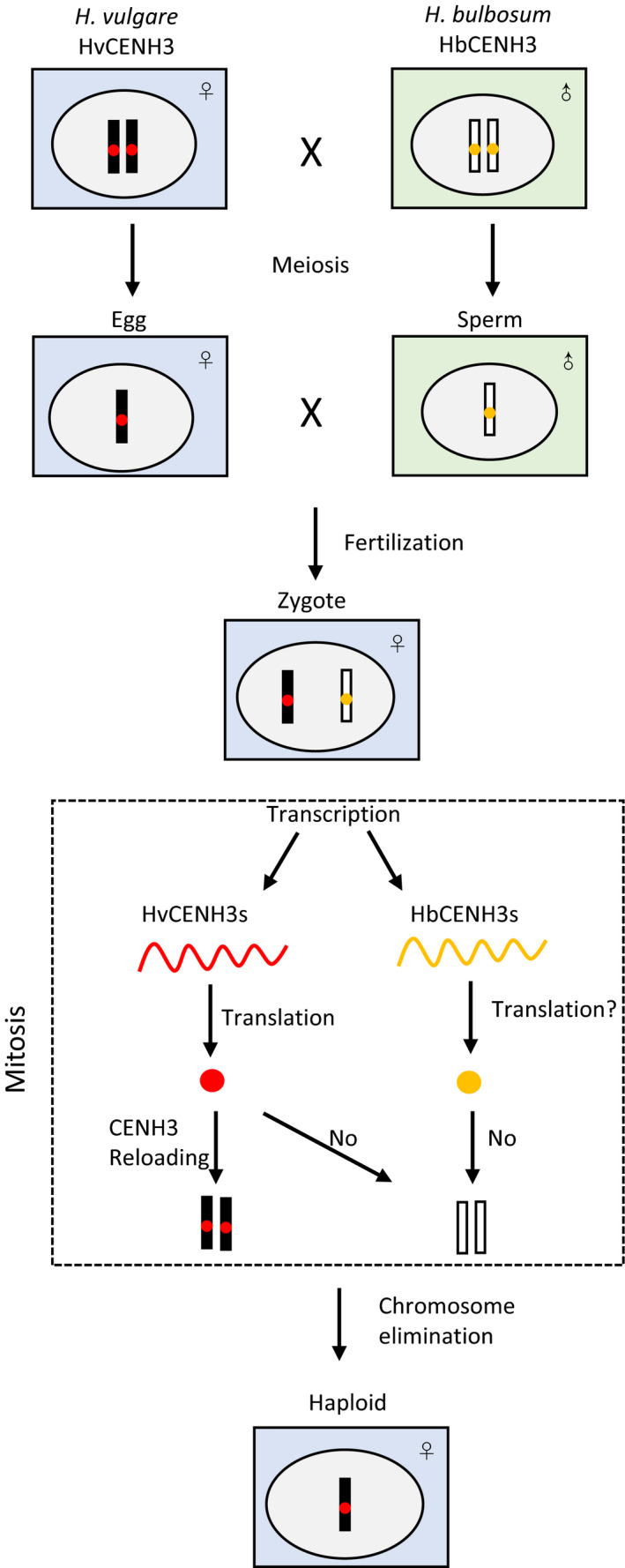
Proposed model of chromosome elimination in *H*. *vulgare* × *H*. *bulbosum*.

Sanei *et al*. (2011) studied the mechanism during the early development of *H. vulgare* × *H. bulbosum* and found that CENH3 plays an important role in chromosome elimination (Figure 1). CENH3 (CENP‐A in humans), a histone H3 variant that replaces standard histone H3 in centromeric nucleosomes, determines the position of centromeres and is necessary for chromosome segregation during cell division (Britt and Kuppu, 2016; Ravi and Chan, 2010). Many proteins are involved in CENH3 loading and assembly, any error of which would result in nonfunctional centromeres (Allshire and Karpen, 2008; Sanei *et al*., 2011; Topp *et al*., 2009). In unstable *H. vulgare* × *H. bulbosum* hybrids, transcription of all CENH3 genes in both parents occurs after fertilization. HvCENH3 has translation activity and can be loaded properly to the centromeres of *H. vulgare*, but whether HbCENH3 has translation activity is unknown. The *H. bulbosum* centromere has no activity during anaphase leading to chromosome elimination and *H. vulgare* haploid embryo development (Watts *et al*., 2016). Centromere inactivity attributes to centromeric loss of CENH3 protein instead of uniparental transcription inactivation of CENH3 genes (Sanei *et al*., 2011).

### Haploidization via intraspecific hybridization

Haploid production by intraspecific hybridization is the predominant way in maize (Chang and Coe, 2009; Geiger, 2009). This method was first reported in 1959 with the discovery of maize haploid inducer Stock 6, producing 2%–3% maternal haploids when outcrossed as a male. Over the years, haploid induction rates (HIR) increased to 8%–10% due to development of high inducing lines, such as WS14, RWS, UH400, BHI306 and CAU5 (Wu *et al*., 2014; Xu *et al*., 2013). At present, haploid identification largely relies on dominant marker gene R1*‐nj* (purple scutellum and aleurone). For rapid discrimination of haploid progeny from diploid seed, Liu *et al*. (2012) proposed a high‐throughput system based on kernel oil content by pollination with high oil inducer lines (Melchinger *et al*., 2013; Wang *et al*., 2016).

Genes and QTL involved in maternal haploid induction in maize have been reported in many recent studies. Barret *et al*. (2008) detected a major locus (*ggi1*) on chromosome 1 causing *in situ* gynogenesis and segregation distortion (SD). Their results showed that the pollen genotype determines its ability to induce haploid female embryos. Prigge *et al*. (2012) conducted a QTL analysis for HIR in four populations including two haploid inducer lines, CAUHOI (HIR = 2%) and UH400 (HIR = 8%). Eight QTL have been identified with two large‐effect QTL *qhir1* and *qhir8* on bins 1.04 and 9.01, respectively, explaining up to 66% and 20% of the genetic variance. The *qhir1* region also showed high SD against the inducer haplotype suggesting that haploid induction ability is associated with failure to transmit inducer gametes. Xu *et al*. (2013) narrowed down the genome region responsible for SD (*sed1*) to a 450‐kb region. They assumed that the *sed1* locus causes epigenetic and dosage‐dependent modification of chromosomes. The different *sed1* expressions among pollen grains from *sed1/sed1* plants result in chromosome epigenetic modifications. A weak modification in chromosomes of sperm cell will result in the formation of normal diploids. In contrast, a strong modification will lead to haploid formation or kernel abortion. Dong et al. (2013) narrowed down the region of *qhir1* into 243 kb region. Kelliher *et al*. (2017) found that haploid induction in maize is a postzygotic character attributed to a frame‐shift mutation in *MATRILINEAL* (*MTL)* (also called *ZmPLA1* and *NLD*), which was identified by fine mapping, genome sequencing, genetic complementation and gene editing. MTL is a phospholipase specific to the sperm cell cytoplasm. Novel edits in *MTL* result in 6.7% haploid offspring. A 4‐bp (CGAG) insertion in the fourth exon of *ZmPLA1* in CAU5 (a haploid inducer derived from Stock 6) compared to the B73 reference genome was shown to be the cause of the haploid induction phenotype using CRISPR/Cas9 gene editing (Liu *et al*., 2017). In the *ZmPLA1* knockout lines, the average HIR is approximately 2%, which is close to the HIR of stock 6, indicating that the *ZmPLA1* knockout method can be used to create haploid inducers. *MTL* is highly conserved in cereals, and those two findings may contribute to the development of intraspecific *in vivo* haploid induction systems in crop plants without existing efficient DH technology. HIR relies not only on haploid inducers but also on maternal genetic background. Wu *et al*. (2014) identified two QTL on chromosome 1 (*qmhir1*) and chromosome 3 (*qmhir2*), which highly affect haploid induction from the maternal side, explaining 14.7% and 8.4% of the genetic variation, respectively.

**Figure 2:**
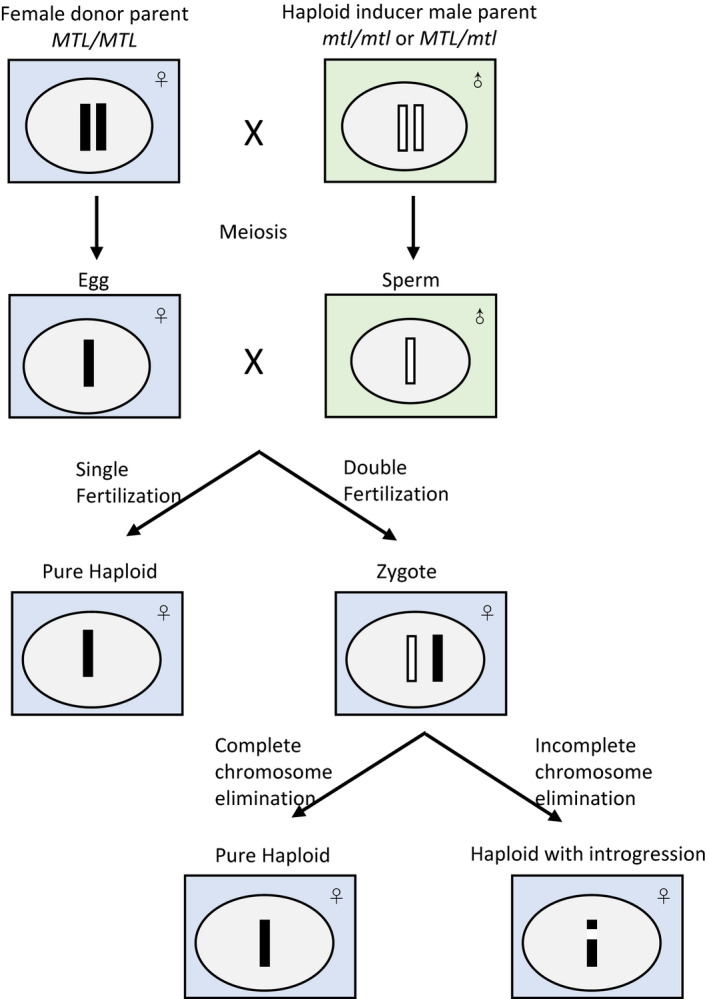
Two possible mechanisms of *in vivo* haploid induction in maize.

Two hypotheses, single fertilization and postzygotic genome elimination (Figure 2), have been presented for the *in vivo* haploid induction mechanism of maternal haploids in maize (Sarkar and Coe, 1966; Zhao *et al*., 2013). For the single fertilization hypothesis, the failure fusion of sperm and egg causes haploid embryogenesis. In case of genome elimination, the inducer's chromosomes are eliminated after normal double fertilization. Li *et al*. (2009) induced maize ZD958 by pollination with a high oil inducer line CAUHOI to produce maternal haploids. About 43.18% of the diploid‐like haploids carrying CAUHOI chromosome segments and the introgressed CAUHOI genome in haploids are small (1.79%–2.92%). Two new maize inducers, CAU^B^ containing B chromosomes and CAU^YFP^ containing CENH3‐YFP, were developed to investigate the mechanism of haploid induction by Zhao *et al*. (2013). B chromosomes were detected in a few haploids and a ~44‐Mb inducer fragment was found in a single haploid, indicating that haploid formation involves double fertilization. Chromosome elimination starts at the very beginning of embryonic development, which was believed to be associated with the functional defects of the CENH3 gene in interspecific hybridizations (Sanei *et al*., 2011). However, there are no differences in the coding sequence and mRNA expression levels of the CENH3 gene between inducers and noninducers, suggesting that CENH3 does not contribute to *in vivo* maternal haploid induction in maize. In the cross HZ514 (sweet corn) × HZI1 (inducer), mosaic endosperm kernels, mixploidy and aneuploidy were observed Qiu *et al*. (2014). Around 7.37% of the haploids contained HZI1 segments. Taken together, all these results suggest that uniparental chromosome elimination leads to the formation of haploid, but the possibility that single fertilization might contribute to haploid induction cannot be excluded. Considering *MTL* has been identified as the key gene contributing to haploid induction, careful embryology after *MTL*‐mediated haploid inducer pollinations and quantitative data tracking the male DNA in haploids are required to clarify the precise mechanism of haploid induction in maize (Kelliher *et al*., 2017; Liu *et al*., 2017; Qiu *et al*., 2014; Zhao *et al*., 2013).

### Haploidization via centromere‐mediated chromosome elimination

Over the last century, all the technologies discussed above have proven to be useful in haploid induction, but they are limited to particular crop genotypes and species. Ravi and Chan (2010) described a novel method of *in vivo* haploid induction through centromere‐mediated genome elimination in Arabidopsis based on CENH3. CENH3 consists of an N‐terminal tail region, which is highly variable even between closely related species, and a C‐terminal Histone Fold Domain (HFD), which is well conserved across species (Britt and Kuppu, 2016; Kuppu *et al*., 2015; Malik and Henikoff, 2003). The N‐terminal tail has one alpha helix (αN), and the HFD domain has three alpha helices separated by two loop regions (α1‐L1‐α2‐L2‐α3) (Ishii *et al*., 2015; Watts *et al*., 2016). The CENP‐A targeting domain (CATD) composed of loop 1, and α2 is necessary for CENH3 loading to the centromere (Black *et al*., 2007; Lermontova *et al*., 2006; Sullivan *et al*., 1994).

Ravi *et al*. (2010) found that transgenic green fluorescent protein‐tagged (GFP) CENH3 (*GFP‐CENH3*) was able to complement the phenotype of *cenh3‐1*, an embryo‐lethal null mutant, although some lines showed reduced complementation. The results indicate that the GFP tag affects the function of centromeres. Further, to construct a chimeric *H3.3/CENH3* protein, the N‐terminal tail of CENH3 was replaced by the tail of a conventional Arabidopsis histone H3. Then, the protein fused with a GFP reporter to construct the *GFP‐tailswap* protein, which could rescue cenh3‐1 mutants (Ravi and Chan, 2010). However, *GFP‐tailswap* plants were mostly male sterile, attributable to meiosis defects. After self‐pollination, *GFP‐tailswap* plants produced normal diploid seed at about 1% of the normal rate (Ravi and Chan, 2010; Ravi *et al*., 2011). On outcrossing, the chromosomes from *GFP‐tailswap* were frequently lost postfertilization producing haploids and aneuploids. When crossed with wild‐type plants as female parent, *GFP‐tailswap* plants produced 25%–45% maternal haploids and 28%–50% aneuploids. These rates were reduced to 4%–5% for paternal haploids and 4%–11% for aneuploids when the line is used as male parent. Both the maternal and paternal haploids contained the chromosomes from the wild‐type parent and the cytoplasm from the maternal parent. Haploids were generally sterile, but did produce some DH seed spontaneously by meiotic nonreduction. Not only *GFP‐tailswap* plants can produce haploids, but also *GFP‐CENH3* plants can produce 5% haploids when crossed to wild‐type plants through chromosome missegregation. Ravi and Chan (2010) proposed that the function of modified centromeres is normal unless when they are constrained to have a competition with wild‐type centromeres for centromere loading. As all crop species have CENH3, haploids induced by CENH3 modification may be extended to other crops. Application of this new method—chromosome elimination—to other crops requires two steps, knocking out or down the native CENH3 gene and complementing the native CENH3 with an altered CENH3 (Comai, 2014).

Ravi *et al*. (2014) found that the Arabidopsis *GFP‐tailswap* plants not only produce Arabidopsis haploids (*n *=* *5) but also produce *A. suecica* haploids (*n *=* *13). When pollinating Arabidopsis *GFP*‐*tailswap* plants with allopolyploid species *A. suecica* pollen, two of 241 viable progenies were identified as *A. suecia* haploids. Thus, haploid inducers produced in one species could be applied for haploid induction in its closely related species via interspecific chromosome elimination.

Maheshwari *et al*. (2015) asked whether natural variants in CENH3 sequences affects chromosomal segregation in zygotic mitosis of hybrids by complementation tests using the Arabidopsis *cenh3‐1* mutant, with untagged CENH3s from a variety of plant species. They found that natural variation of CENH3, even from the monocot maize CENH3, can complement *cenh3‐1* mutant indicating that the basic function of CENH3 is well conserved. Transgenic CENH3 plants are self‐fertile but produce haploids and aneuploids on outcrossing. Haploids inherit only the chromosomes carrying the wild‐type CENH3. Furthermore, they replaced the Arabidopsis HFD domain with the *L. oleraceum* HFD domain (AtNTT‐LoHFD) and found that transgenic CENH3 plants perform normally when crossed with wild‐type lines. However, transgenic CENH3 plants (LoNTT‐AtHFD) induce haploids which indicate that variation in the N‐terminal tail of CENH3 leads to segregation errors.

To clarify whether minimal mutations in HFD of CENH3 affect centromere function, Karimi‐Ashtiyani *et al*. (2015) tested the function of mutated CENH3s in a barley population developed by TILLING. They found that a single‐point amino acid substitution in HFD impairs CENH3 loading to the centromere, such as L92F in barley, L106I or L106F in sugar beet and L130I or L130F in Arabidopsis. When pollinated with wild‐type plants, *Atcenh3* L130F‐1 induced haploids and aneuploids which may attribute to the less total CENH3 protein than wild‐type plants. In parallel, Kuppu *et al*. (2015) conducted complementation tests on *cenh3‐1* with a variety of mutant CENH3s that each has single amino acid substitution in HFD conserved residues. The mutant CENH3s with single amino acid changes P82S, G83E, A132T, A136T and A86V exhibited no significant effect in the process of meiosis or mitosis and gave normal progenies on self‐pollination, while inducing postzygotic incompatibility and low frequency of paternal haploids when outcrossing to wild‐type plants. As a specific amino acid mutation in HFD is sufficient to generate haploid inducers, a simple one‐step method, EMS‐mediated mutagenesis, has been proposed for the development of nontransgenic haploid inducer. The rapid progress of genome editing comprising ZFNs, TALENs and CRISPR‐Cas9 also enables to manipulate CENH3 to develop haploid inducers.

To check whether haploid induction via centromere‐mediated genome elimination can be engineered in other crops, *AcGREEN‐tailswap‐CENH3* and *AcGREEN‐CEHN3* transgenes were used to complement the phenotype of CENH3 knockout and knock‐down lines in maize (Kelliher *et al*., 2016). In the CENH3 knock‐down (RNAi) strategy, *AcGREEN‐tailswap‐CENH3* lines produced an average of 0.16% haploids, while *AcGREEN‐CENH3* lines rarely produced haploids. In the CENH3 knockout strategy, 0.23% and 0.14% haploids were induced on average by *AcGREEN‐CENH3* hemizygous and homozygous lines, and 0.53% and 0.13% haploids were induced by *AcGREEN‐tailswap‐CENH3* hemizygous and homozygous lines, respectively. The highest haploid induction rate reached 3.6% in several *AcGREEN‐tailswap‐CENH3* hemizygous lines when backcrossed as male parents. Although the haploid induction rate is too low to match maize commercial inducers, this is the first report of haploidization via CENH3‐mediated chromosome elimination in maize and will promote the generation of haploid inducers in other species (Watts *et al*., 2016).

Watts *et al*. (2017) studied CENH3 engineering for haploid inducer development in *Brassica juncea*, a polyploid crop carrying three copies of CENH3 resulting in five different transcripts. They knocked down the native CENH3 genes using an RNAi method and then *GFP‐CENH3‐tailswap* plants were used to rescue the CENH3 silenced cells. Cotransformed plants carrying both silencing and rescue constructs resulted in normal seed set after selfing. However, when cotransformed lines were crossed to untransformed lines, many aneuploids and one haploid were identified from a total of 140 progenies. These results indicate that CENH3 engineering and RNAi can be used to generate haploid inducers in polyploid crops.

One of the key characteristics of centromere‐mediated chromosome elimination is the production of aneuploid. There are three types of aneuploid chromosome: (i) containing an extra copy of an entire chromosome; (ii) carrying an additional truncated chromosome; and (iii) having an extra copy of shattered chromosome (Britt and Kuppu, 2016; Ishii *et al*., 2016; Tan *et al*., 2015). Shattered chromosomes originated from the parent with a mutant *CENH3,* and some of them can be sufficiently stable to be inherited. By nonhomologous end joining (NHEJ), the DNA Ligase 4 enzyme, which specifically repairs double‐strand breaks, is involved in the shattered chromosomes recombination (Tan *et al*., 2015). Crossing *GFP‐tailswap* or *lig4‐2 GFP‐tailswap* lines by *lig4‐2/lig4‐2* mutants enhanced haploid induction rates at the expense of both diploids (from 40% to 81%) and aneuploids (from 39% to 83%). Parent‐specific haploidization can thus attribute to early loss of the wild‐type *LIG4* allele located on inducer chromosomes, and formation of diploids and aneuploids results from LIG4‐dependent chromosome rescue. In mammalian systems, the frequency of uniparental chromosome elimination can be increased by unrepaired DNA damage (Wang *et al*., 2014). Overall, DNA repair mutants may be used to increase haploid induction rates in the CENH3‐mediated genome elimination system (Britt and Kuppu, 2016).

To identify haploid seed pregermination, Ravi *et al*. (2014) developed an improved haploid inducer, SeedGFP‐HI, by introducing GFP expressed under the control of promoter 2S3 (the seed storage protein) (At2S3: GFP) (Kroj *et al*., 2003) into *GFP*‐*tailswap* plants. This visible marker is in the endosperm and embryo. F_1_ seed derived from SeedGFP‐HI crosses showed two classes of phenotypes: (i) uniformly fluorescing seed (GFP expressed both in endosperm and embryo); (ii) mottled GFP seed (GFP only expressed in endosperm instead of embryo). All uniform seed consists of diploids and aneuploids, while 91% of the mottled GFP seed was haploid and the rest aneuploid. Thus, preselection of mottled GFP seed increases early haploid selection efficiency.

**Figure 3:**
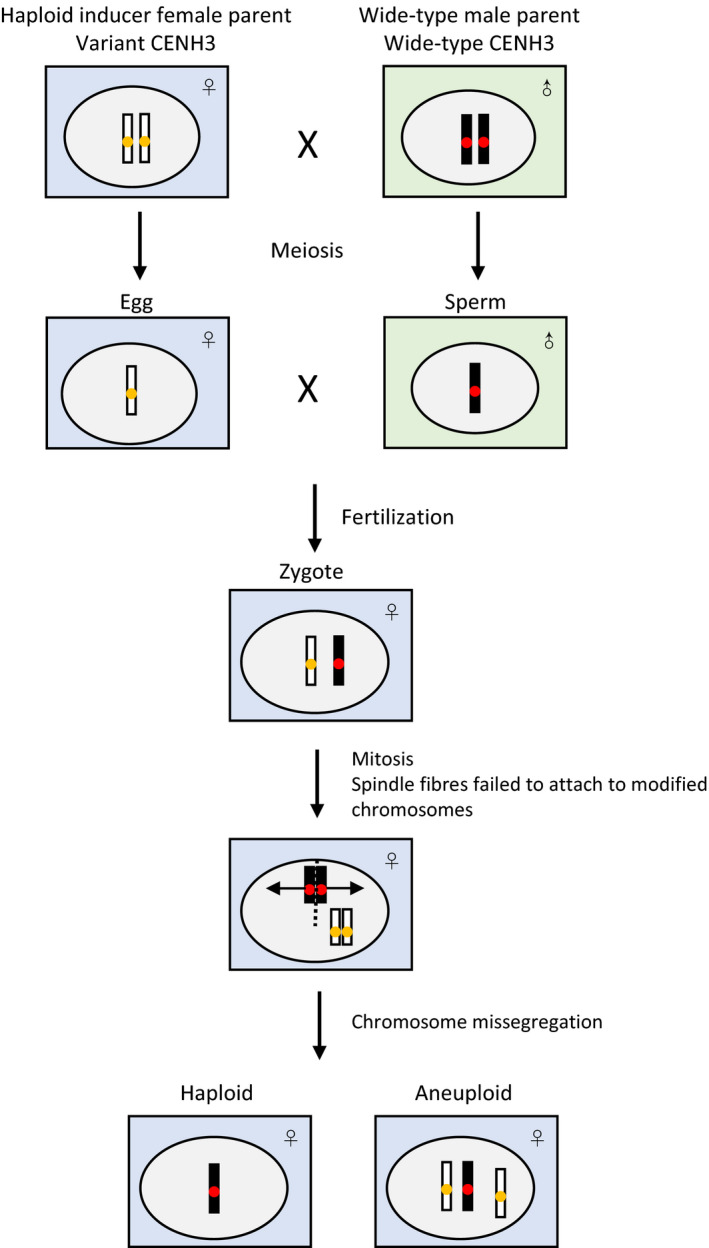
A model for the process of haploid induction via modification of CENH3.

The possible mechanism of centromere‐mediated chromosome elimination is shown in Figure 3. The addition of a bulky tag and sequence changes of CENH3 can affect protein function for CENH3 reloading (Britt and Kuppu, 2016; Watts *et al*., 2016). The modified centromere behaves normally, while being less competitive to wild‐type centromeres. Both maternal and paternal CENH3s are actively removed from the F_1_ zygote nucleus within 2–4 h after fertilization, and reloading occurs in the F_1_ zygote within 6–8 h after fertilization (Ingouff *et al*., 2010). Chromosomes bearing modified CENH3 showed centromere reloading impair or delay leading to the loss of centromere function (Ishii *et al*., 2016). As a result, spindle fibres failed to attach to chromosomes of haploid inducers which may be lost completely or fail in segregation, but remain in one daughter leading to development of haploids and aneuploids (Dwivedi *et al*., 2015; Watts *et al*., 2016).

## Genome doubling

### Artificial genome doubling

Artificial genome doubling is the most popular method applied for doubling the genomes in large‐scale DH line production. Colchicine, an antimicrotubule drug, has been widely used and is the most effective genome doubling agent. Colchicine duplicates the genomes by binding to tubulins to inhibit microtubule polymerization (Kleiber *et al*., 2012; Prasanna *et al*., 2012; Wan *et al*., 1989; Weber, 2014). However, colchicine is highly toxic which is not only potentially carcinogenic but also hazardous to the environment (Melchinger *et al*., 2016). The effects of other agents with lower toxicity on chromosome doubling, such as amiprophos‐methyl (APM), oryzalin, pronamide and trifluralin, all of which are herbicides, have been reported (Beaumont and Widholm, 1993; Häntzschel and Weber, 2010; Murovec and Bohanec, 2012; Wan *et al*., 1991). The haploid genome doubling rate of trifluralin treatment in *B. napus* was 85.7%, for colchicine 74.1% and for oryzalin 66.5%, compared to only 42.3% without any treatment (KlíMa *et al*., 2008). APM combined in an optimum dosage with pronamide has similar rates of genome doubling as colchicine in maize (Melchinger *et al*., 2016). Kato and Geiger (2002) developed an effective genome doubling procedure in maize using a nitrous oxide (N_2_O) gas treatment at the six‐leaf stage, with about 44% of the haploids producing seed after selfing.

### Spontaneous genome doubling

Spontaneous genome doubling has been reported in several species. The frequency of spontaneous genome doubling is 10%–40% in *Brassica napus*, 70%–90% in barley, 50%–60% in rice, 50%–90% in rye and 25%–70% in bread wheat (Castillo *et al*., 2009; Henry, 1998; Seguí‐Simarro and Nuez, 2008). In a species, the doubling rate oscillates enormously among genotypes (Chalyk, 1994; Kleiber *et al*., 2012). Kleiber *et al*. (2012) reported that the range of haploid fertility is 0%–20% in tropical and temperate maize germplasm. Wu *et al*. (2016) reported that haploid male fertility ranges from 9.8 to 89.8%. The maize inbred line ‘Yu87‐1’ showed the highest male fertility. Ren *et al*. (2017) detected four QTL controlling haploid male fertility and fine‐mapped the key QTL *qhmf4* on chromosome 6. The candidate gene of *qhmf4* is the absence of first division 1 (*AFD1*), which is required for axial element elongation. In the *afd1* mutant, the meiotic first division is replaced by an equational division. Marker‐assisted selection (MAS) can be used to improve haploid fertility.

Improved chromosome doubling can also be obtained by mutation. In Arabidopsis, fertile haploids can be obtained by combining three mutants: *osd1, rec8* and *spo11‐1* (Cifuentes *et al*., 2013). OSD1 controls the conversion from meiosis I to meiosis II, and both SPO11‐1 and REC8 are required in key meiotic process. This genotype is called *MiMe* and transfers meiosis into mitosis. Sugihara *et al*. (2013) induced a *first division restitution* (*fdr1*) mutant by sodium azide treatment. *fdr1* haploids restore haploid male fertility attributable to first division restitution and produce diploid kernels.

### Combing haploids with minichromosomes

Genetic engineering with a few genes, such as herbicide resistance genes and *Bacillus Thuringiensis* (Bt) toxin genes, has changed agriculture by increasing crop yield and reducing the use of pesticides in the last 20 years (Yu *et al*., 2016). The next generation of genetic engineering must depend on the transfer of multiple genes, which is required for complex traits in plant biotechnology, and is still difficult to achieve (Halpin, 2005; Yu *et al*., 2016). The development of minichromosome technology, providing a super vector platform, offers a new approach to genetic engineering with multiple genes. Minichromosome is engineered chromosome that remains stable in the process of meiosis and mitosis and does not engage in recombination with other chromosomes. When used as a vector for expressing foreign genes, it has little effect on the growth and development of the host (Acevedo‐Garcia *et al*., 2013). There are usually two methods for the construction of minichromosomes: ‘bottom‐up’ by assembling all essential parts, like centromere repeats, telomeres and replication origins, or ‘top‐down’ by chromosome truncation. The bottom‐up method has been successfully used in mammalian and yeast cells (Harrington *et al*., 1997; Murray and Szostak, 1983), but its application in plants continues to be an ongoing debate (Gaeta *et al*., 2011; Houben *et al*., 2008; Mette and Houben, 2015). In contrast, minichromosome construction by bottom‐up methods has been well established in plants (Birchler, 2014). The use of supernumerary or B chromosomes in maize is a good choice for minichromosome construction, because they are nonessential and have little effect on phenotypes until their copy numbers approach 15 (Birchler, 2014). When minichromosomes are constructed that meet all the demands of a suitable vector, the insertion of multiple expression cassettes will be needed. Site‐specific recombination (SSR)‐mediated method can be used in combination of gene assembly technology to stack transgenes in minichromosomes (Yu *et al*., 2016).

Minichromosomes are usually assembled in certain genetic backgrounds and then transferred to other genotypes by repeated backcrossing for practical applications or functional assessment of transgenes in different genetic backgrounds. Recent studies suggest that haploid breeding can facilitate minichromosome transfer to different genetic backgrounds. Zhao *et al*. (2013) discovered that the B chromosome of a maternal haploid inducer line can be transferred to haploid progenies as an extra chromosome in maize. In oat × maize crosses, not only oat haploids but also oat–maize chromosome addition lines were generated, where a haploid oat genome carrying an extra maize chromosome were generated (Ananiev *et al*., 1997; Jin *et al*., 2004). Although alien chromosome addition lines construction has not been well studied in the centromere‐mediated haploidization systems, better understanding of the mechanism of centromere‐mediated chromosome elimination may give new ideas for the construction of alien chromosome addition line. It is possible that minichromosomes present in inducers can be transferred to haploids (Birchler, 2014). If successful, the time required to transfer minichromosomes to new inbred lines in conjunction with haploid induction is reduced compared to repeated backcrossing. If minichromosomes contain genes controlling spontaneous haploid genome doubling, introgressing minichromosomes into inducer lines would result in ‘Super Haploid Inducers’ which can not only induce haploids but also increase haploid fertility. Breeders could in this case avoid the use of hazardous chemicals, especially colchicine, by directly using super haploid inducers to produce DH lines.

## Applications of haploids in plant breeding and genetic analysis

### Exchanging cytoplasmic and nuclear genomes

In Arabidopsis, both maternal and paternal haploids containing wild‐type chromosomes and maternal cytoplasm can be generated using CENH3‐mediated haploid inducers as the male or female parent. Ravi *et al*. (2014) developed a *cenh3‐1 GFP‐tailswap* haploid inducer with Ler cytoplasm, Ler‐cytoplasmic haploid inducer (HI). When pollinating Ler‐cytoplasmic HI with pollen from wild type with Col‐0 cytoplasm, Col‐0 WT, haploids with Col‐0 WT chromosomes and Ler cytoplasm are generated. This method can be used to develop any combination of cytoplasmic and nuclear genomes by transferring the male nuclear genome into a heterologous cytoplasm rapidly and conveniently. This facilitates the production of new cytoplasmic male sterile (CMS) lines for F_1_ hybrid seed production. If the haploid inducer line has CMS background, pollinating this haploid inducer line with different inbred lines generates paternal haploids, which carry CMS. One or a few paternal haploids need to be pollinated with pollen from the maternal inbred to produce a new diploid CMS line. Using paternal haploids for cytoplasmic conversions has three distinct advantages: (i) only two generations are needed; (ii) the new CMS line has 100% of the genomes of the inbred line; and (iii) chromosome doubling is not required (Weber, 2014). This method has been employed in maize using the *ig1* system for quite a while (Evans, 2007).

### Reverse breeding

Hybrid seed is traditionally produced from a cross between two inbred lines. Dirks *et al*. (2009) proposed a novel plant breeding technology, reverse breeding, which can directly generate parental inbred lines from any hybrid. There are three steps required for reverse breeding: (i) inhibition of meiotic crossover in F_1_ plants to produce gametes containing combinations of nonrecombinant parental chromosomes, (ii) generation of DH lines via in vitro unfertilized ovule or anther culture and (iii) regeneration of the original hybrid through crossing DH lines with complementary sets of parental chromosomes.

Reverse breeding has been tested in Arabidopsis by Wijnker *et al*. (2012). Firstly, they crossed Landsberg (Ler‐0) and Columbia erecta (Col‐0) to develop an F_1_ hybrid. In the hybrid, the meiosis crossover is suppressed using RNAi to knock‐down the DMC1 gene, which is required for the crossover formation during meiosis. Secondly, they crossed this hybrid to the centromere‐mediated haploid inducer line to generate haploids which were doubled into DH lines through spontaneous doubling. Genetic analysis of 69 DH lines using SNP markers at approximately 4‐Mb intervals showed absence of recombination. Lastly, they recovered the original hybrid by crossing complementing DH lines. Wijnker *et al*. (2014) proposed a procedure of reverse breeding in five steps: (i) the generation of *DMC1:RNAi* transgenic lines (achiasmatic parental lines); (ii) development of achiasmatic hybrids; (iii) haploid induction by crossing to *GFP‐tailswap*; (iv) generation of DH lines by self‐pollination of haploids; and (v) recreation of original hybrids by crossing DH lines with complementary sets of parental chromosomes. Successful reversing of breeding in Arabidopsis and availability of centromere‐mediated haploid induction technology make it possible to apply this technology to other crops.

### Gene stacking from biparental crosses

Nowadays, introgression of one or a limited number of genes into elite inbreds by marker‐assisted backcrossing is routine in plant breeding (Lübberstedt and Frei, 2012). At the end of backcross programmes, a heterozygous plant is selfed to produce a fixed line. For single gene introgression, the expected probability of individuals with desired homozygous genotype is 1/4. The frequency of expected genotypes decreases exponentially following the formula 1/4^*n*^, where *n* is the number of independently segregating genes (Lübberstedt and Frei, 2012; Ravi *et al*., 2014; Shen *et al*., 2015). In contrast, haploid target genotypes are generated with a frequency of 1/2^*n*^. For example, for five loci, the frequency of the desired homozygous genotype is 1/1024 in selfed diploid progeny and 1/32 in haploid progeny. Application of doubled haploids thus significantly reduces the population size required to find desirable genotypes.

### Accelerate plant breeding by MAS and GS

The availability of cheap and abundant molecular markers allows breeders to apply MAS and genomic selection (GS) in crop improvement. MAS depends on the identification of markers significantly associated with the trait. MAS allows breeder to discard a large number of plants with undesired gene combination, pyramid beneficial genes in subsequent generations, minimize field testing and reduce the number of generations (Collard and Mackill, 2008; Dwivedi *et al*., 2015). The combination of MAS and DHs offers new opportunities for increasing genetic gain and shortens the time required to cultivar breeding. MAS and DH have been successfully used to accelerate resistance breeding in cereal crops. Wessels and Botes (2014) developed a series of DH wheat lines containing rust resistance genes. MAS was used for the selection of resistance genes, and DH technology was used for the generation of homozygous lines. This study demonstrated that integration of MAS and DH technology into conventional breeding processes can increase the speed of cultivar development.

In contrast to MAS, GS employs genomewide markers to predict genomic estimated breeding values (GEBVs) (Daetwyler *et al*., 2015). GS requires a training population to estimate GEBVs based on phenotypic and genotypic data. In a breeding cycle, the estimated marker effects in the training population can then be used for GEBVs prediction without phenotyping (Heffner *et al*., 2009). As an individual's GEBV can be predicted before or without phenotypic characterization, this enables breeders to make early selection decisions which could increase genetic gains and shorten breeding cycles (Daetwyler *et al*., 2015). Mayor and Bernardo (2009) studied GS and MAS in DH versus F_2_ populations and found that GS was superior to MAS and DH populations are superior to F_2_‐derived population using GS.

## Summary and outlook

Doubled haploid technology has been successfully used in crop improvement and genetic analysis. Numerous studies have provided a better understanding of the genetic of haploid induction. In interspecific hybridization, chromosome elimination is associated with the uniparental centromere inactivation (Sanei *et al*., 2011). In intraspecific hybridization, *MTL*, a sperm‐specific phospholipase, is proved to be the cause of haploidization in maize (Gilles *et al*., 2017; Kelliher *et al*., 2017; Liu *et al*., 2017). Conservation of MTL in cereals may enable the development of intraspecific *in vivo* haploid inducer lines in crop plants to accelerate plant breeding. The report of haploidization by CENH3‐mediated chromosome elimination in Arabidopsis is a breakthrough in haploid induction. This method has been demonstrated in maize (monocotyledonous crop) and *Brassica juncea* (polyploid crop) indicating that it could be applied in other crops. The combination of haploid induction and minichromosome offers a new strategy for introducing multiple genes into elite lines. One possible application is the development of ‘Super Haploid Inducers’, which lead to haploids with spontaneous haploid genome doubling capability. Integrating DH technology with MAS and GS offers new insights to minimize breeding cycles and maximize genetic gains. DH technology is useful in reverse breeding, CMS line production, gene stacking and a variety of other applications.

Although many of the technological problems of DH technology have been overcome, challenges still exist in application of DH technology. Understanding of the mechanism of haploid induction is still incomplete. It is unclear, whether the mechanism of *in vivo* haploid induction in maize is chromosome elimination or single fertilization. In centromere‐mediated haploid induction systems, the mechanism of CENH3 modification affecting only chromosome segregation by outcrossing on wild‐type plants, without affecting self‐pollination, remains unknown. By a better understanding of the process of haploid induction, it is possible to improve haploid induction systems and deliver DH technology into other crops, such as banana and cassava. Only few studies have been reported on the genetics and mechanism of spontaneous haploid genome doubling, which is important to avoid the use of hazardous chemicals.
